# Morphometric, densitometric and mechanical properties of mandibular deciduous teeth in 5-month-old Polish Merino sheep

**DOI:** 10.1186/1746-6148-10-45

**Published:** 2014-02-19

**Authors:** Marcin R Tatara, Anna Szabelska, Witold Krupski, Barbara Tymczyna, Iwona Łuszczewska-Sierakowska, Jarosław Bieniaś, Monika Ostapiuk

**Affiliations:** 1 Department of Animal Physiology, Faculty of Veterinary Medicine, University of Life Sciences in Lublin, ul. Akademicka 12, 20-950 Lublin Poland; 2 II Department of Radiology, Medical University in Lublin, ul. Staszica 16, 20-081 Lublin, Poland; 3 Department of Prosthetic Dentistry, Medical University in Lublin, ul. Karmelicka 7, 20-081 Lublin, Poland; 4 Department of Conservative Dentistry and Endodontics, Medical University in Lublin, ul. Karmelicka 7, 20-081 Lublin, Poland; 5 Department of Animal Anatomy, Faculty of Veterinary Medicine, University of Life Sciences in Lublin, ul. Akademicka 12, 20-950 Lublin, Poland; 6 Department of Materials Engineering, Lublin University of Technology, ul. Nadbystrzycka 36, 20-618 Lublin, Poland

**Keywords:** Sheep, Teeth, Densitometry, Quantitative computed tomography, Microtomography

## Abstract

**Background:**

Caries, enamel hypoplasia, molar incisor hipomineralization, amylogenesis imperfecta, dentine dysplasia, hypophosphatasia and other dental disorders lead to tooth mineralization disturbances and structural abnormalities, decreasing masticatory organ functions. Dental disorders in sheep can lead to premature slaughter before they have attained final stage of their reproductive life and induce economic loss due to high flock replacement costs. Growth rate, health status and meat quality of sheep depends on tooth properties and quality determining in large extent efficiency of the masticatory apparatus and initial food break up. Considering lack of basic anatomical and physiological data on teeth properties in sheep, the aim of the study was to evaluate morphometric, densitometric and mechanical traits of deciduous mandibular incisor, canine and the second premolar obtained at the slaughter age of 5 months of life.

**Results:**

The obtained results have shown the highest values of weight, total tooth volume, enamel volume and dentine volume in second premolar. Morphometric and mechanical parameters of incisors reached the highest values in first incisor and decreased gradually in second and third incisor, and in canine. Densitometric measurements have not revealed significant differences of the volumetric tooth mineral density in hard dental tissues between the investigated teeth.

**Conclusions:**

In conclusion, proposed methodological approach is noninvasive since the deciduous teeth undergo physiological replacement with permanent teeth. Deciduous teeth can be easy collected for analyses from large animal population and may reflect mineral status and metabolism resulting from postnatal growth and development of the whole flock. In individual cases, evaluation of properties of deciduous teeth may serve for breeding selection and further reproduction of sheep possessing favorable traits of teeth and better masticatory system functions.

## Background

Bone, dentine, cementum and enamel are considered as four types of hard tissue. Teeth are the most durable structures of mammalian body [1]. Except for enamel, dentine and cementum represent specialized types of tissue, where type I collagen contributes significantly to the structure. Even though enamel does not arise from connective tissue and does not contain collagen, its formation follows the principles common to the formation of mineralized tissues. Hard dental tissue formation is the result of a process in which specialized cells secrete an organic matrix undergoing mineralization mainly by calcium and phosphates. Among cells, osteoblasts, odontoblasts, cementoblasts and ameloblasts play essential functions in these processes [2].

Dental disorders in sheep can lead to premature slaughter before they have attained final stage of their reproductive life and induce economic loss due to high flock replacement costs. It must be underlined that growth rate, health status and meat quality of sheep depends on tooth properties and quality determining in large extent efficiency of the masticatory apparatus and initial food break up. While incisor disorders reduce dramatically intake of forage, cheek tooth dysfunction has negative impact on food grinding [3,4]. Thus, impaired chewing efficiency resulting from poor tooth health status leads to reduced food intake and the food eaten is digested less efficiently [5,6]. The function of food break-up performed by teeth has particular importance in ruminants, since they have to chew highly fibrous diet to small pieces enough to aid microbial digestion in the rumen.

Etiology of tooth disorders is multifactorial. Defects of the tooth structure may result from soil ingestion and following abrasion combined with chemical attack from acids present in the ingested soil [7,8]. Bacterial impact may lead to production of acids by hydrolysis of the food debris accumulated on the tooth surface and cause caries - demineralization of the hard tissues (enamel, dentin and cementum) and destruction of the organic matrix of the tooth. In young sheep, caries affects mainly incisors and shows up as deep holes at the neck of deciduous incisors or a snap-off at gum level [9]. It was observed that dental caries in 8–12 months old sheep occurs as the result of diet rich in soluble carbohydrates administered in young growing animals [10]. Higher susceptibility to carries may results also from demineralized enamel resulting from molar incisor hypomineralization (MIH). It is a developmental pathology mainly located in the mesio-buccal cusps, starting at the enamel-dentin-junction (EDJ) and continuing towards the enamel surface. Experimental data indicate that the ameloblasts in the hypomineralized enamel are capable of forming an enamel of normal thickness, but with a substantial reduction of their capacity for maturation of enamel [11]. Enamel hypoplasia in sheep changes enamel translucency and displays as 1–2 mm shallow pits on the surface of incisors [12]. It results from disturbed amelogenesis process during tooth development induced by dietary or disease factors in young growing sheep such as feeding grain diets with high phosphorus and low calcium, gastrointestinal parasitism and excessive fluoride ingestion [13-17]. Fluoride intoxication during tooth development also results in tooth fragility, pitting, discolouration and chalkiness of the enamel [10].

Considering lack of basic physiological data on tooth properties in sheep, the aim of the study was to evaluate morphometric, densitometric and mechanical traits of deciduous mandibular incisors, canine and the second premolar - the analyzed teeth had completely developed all their anatomical structures by the slaughter age of 5 months of life.

## Methods

This experiment was conducted in accordance with national guidelines for animal experimentation and the “Guide for the Care and Use of Laboratory Animals”. The experimental procedures used in this study were approved by The Local Ethics Committee on Animal Experimentation of Medical University in Lublin, Poland (Permission number 6/2008 assigned on 15^th^ February 2008).

### Experimental design and sampling procedure

This study was performed on 7 healthy male Polish Merino lambs born physiologically to ewes. Lambs and ewes were kept indoors in pens under standard rearing conditions and provided with drinking water *ad libitum*. Ewes were fed a standard diet. Starting on the 22^nd^ day of life, lambs were fed a commercial concentrate and hay *ad libitum*. The Lambs were weaned at the age of 10 weeks and sacrificed at the age of 5 months of life. Mean final body weight of sheep reached the value of 42 ± 2.3 kg. To perform morphometric, densitometric and mechanical analyses of completely developed deciduous teeth, three incisors (i_1_ – i_3_), canine (fourth incisor – i_4_) and second premolar (p_2_) were isolated from the left part of the mandible. All isolated teeth were cleaned from soft tissues and cementum, weighed and their length was measured. Teeth samples were kept at room temperature in saline solution that was changed regularly until further analyses.

### Quantitative computed tomography of teeth

Using quantitative computed tomography (QCT) technique and SOMATOM EMOTION SIEMENS apparatus (Siemens, Erlangen, Germany) equipped with Somaris/5 VB10B software (version B10/2004A, Volume Evaluation application package), the determination of mean volumetric tooth mineral density (MvTMD) and total tooth volume (Tvol) was performed. Mean vTMD was measured for whole sample, including all the anatomical structures and reflects mean density of all hard tissues in a single tooth. For Tvol and MvTMD determinations, the volume-of-interest was defined at the values between minimum and maximum density of the investigated samples at 0 and 3071 Hounsfield units (HU), respectively [18].

### Microtomography of teeth

**Figure 1:**
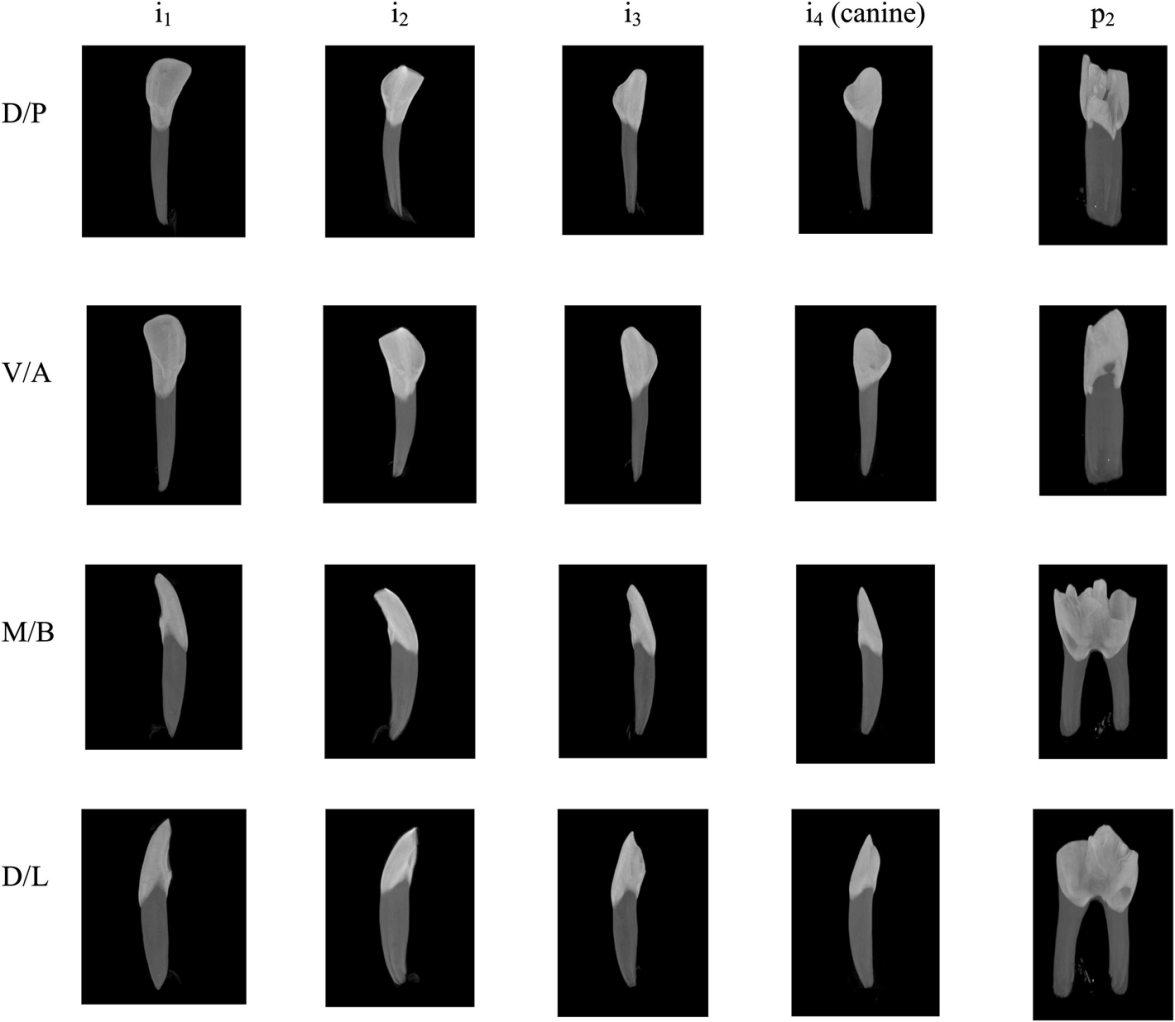
**Three-dimensional microtomography reconstructions of left deciduous teeth in sheep showing the four incisors (i**_**1 **_**– i**_**4**_**) and the second premolar (p**_**2**_**).** The investigated teeth are presented in dorsal/posterior (D/P), ventral/anterior (V/A), mesial/bucall (M/B) and distal/lingual (D/L) views, respectively. The volumetric tooth mineral density (vTMD) was determined in volumes-of-interest (VOIs) separately for whole enamel (volumetric enamel mineral density – vEMD) and for whole dentine (volumetric dentine mineral density – vDMD). Using the same VOIs, total enamel volume (Evol) and total dentine volume (Dvol) were measured automatically for each tooth.

Microtomography (μCT) technique and SkyScan 1174 apparatus (SkyScan n.v., Kontich, Belgium) was used to determine total enamel volume (Evol), volumetric enamel mineral density (vEMD), total dentine volume (Dvol) and volumetric dentine mineral density (vDMD). Images for analysis were obtained at 50 kV and 800 μA with a 0.25 mm aluminum filter. For each tooth, 300 projection images were obtained with a rotation of 0.70°. An image pixel size corresponded to 21.72 μm. The samples were rotated over 180°. Reconstruction of the teeth was performed with the use of NRecon 1.6.1.5 software (SkyScan n.v., Kontich, Belgium). The software allows three-dimensional reconstructions of the investigated sample from the stack of two-dimensional sections (Figure 1). For each slice, the enamel and dentin regions were enclosed using a user-defined polygon. Binary images were obtained with histogram. Two regions of interest (ROI) were defined for the microtomographic images; ROI-1 for enamel and ROI-2 for dentine evaluation [18]. The volume-of-interest was defined at the values between minimum and maximum density of the investigated samples at -1000 and 3071 HU.

### Mechanical analyses of teeth

Mechanical properties of teeth were determined in Zwick/Roell Z010 apparatus (Zwick, Ulm, Germany). Incisors and canines (i_1_ – i_4_) were subjected to a three-point bending test and maximum elastic strength (Wy) and ultimate force (Wf) of the teeth were determined. The distance between tooth supports was set at 40% of tooth length, and the measuring head loaded the investigated samples on the concave (posterior) surface with a constant speed of 10 mm/min. The second premolar tooth (p_2_) was subjected to a compression test and ultimate force (Wf) was determined. Tooth sample was placed on the buccal (lateral) surface and the loading head compressed tooth on the lingual (medial) surface with the speed of 10 mm/min [18].

### Statistical analysis

Statistical analysis was performed using Statistica software (version 6.0 PL, StatSoft, Inc., Tulsa, OK, USA). All data are presented as means ± SEM. The data were found to be normally distributed in accordance with Kolomogorov-Smirnov test. The differences of means between the investigated teeth were tested for statistical significance with the use of one-way ANOVA. Post hoc comparisons of the differences were performed using Duncan’s test. Differences showing *P*-value < 0.05 were considered as statistically significant.

## Results

Results of morphological, densitometric and mechanical properties of the investigated teeth in 5 month old sheep are shown in Table 1. Tooth weight reached the highest value in the second premolar when compared with first incisor, second incisor, third incisor and it reached its lowest value in the canine tooth. Significant differences of the weight were found between all the investigated teeth (P < 0.05). Tooth length reached higher value in first incisor than in second and third incisors while the lowest values of this parameter were found in canine and second premolar. Except for comparison of canine and second premolar showing similar length in both these teeth (P = 0.30), the obtained mean values of this parameter were found to be significantly different (P < 0.001). Total Tvol reached the highest value in the second premolar when compared with the first incisor, second incisor, third incisor and the lowest value in the canine. Significant differences of Tvol were stated between all the investigated teeth (P < 0.05). Total Evol reached significantly higher mean value in second premolar when compared to all the other teeth (P < 0.001). Total Evol of first and second incisors were significantly higher than the value of this parameter measured in the third incisor and canine (P < 0.05). Significant differences of Evol were not found when comparing first and second incisors (P = 0.98) as well as third incisor and canine (P = 0.38). Higher value of total Dvol was found in second premolar than in first and second incisors while the lowest values of this parameter were stated in third incisor and canine. Except for comparison of Dvol of third incisor and canine, where this parameter reached similar values (P = 0.09), the obtained mean values for all the other teeth were significantly different (P < 0.01). Mean volumetric tooth mineral density was not found to be different in the evaluated teeth of 5 month old sheep (P > 0.05). Mean values of vEMD were not significantly different in all the evaluated teeth (P > 0.05). Volumetric dentine mineral density reached significantly higher value in first incisor when compared to this parameter in second and third incisors (P < 0.05). The other comparisons of vDMD have not shown significant differences in the investigated teeth (P > 0.05). Higher value of Wy was stated in the first incisor than in the second and third incisor while the lowest value of this parameter was obtained in canine. Significant differences of Wy were found between all the evaluated teeth (i_1_ – i_4_) in the three-point bending test (P < 0.05). The highest value of ultimate force was obtained executing compression in second premolar when compared to the other teeth (P < 0.001). The three-point bending test of i_1_ – i_4_ revealed higher value of Wf in first incisor than in second and third incisors while the lowest value of this parameter was stated in canine. The differences of Wf in all these teeth were found to be significant (P < 0.05).

**Table 1:** Morphometric, densitometric and mechanical properties of mandibular deciduous incisors, canine and second premolar in 5 month old sheep

**Investigated parameter**	**First incisor**	**Second incisor**	**Third incisor**	**Fourth incisor (canine)**	**Second premolar**
Weight (mg)	369^a^ ± 10	290^b^ ± 6	199^c^ ± 4	150^d^ ± 3	783^e^ ± 38
Length (mm)	28.00^a^ ± 0.48	25.57^b^ ± 0.48	22.28^c^ ± 0.18	19.00^d^ ± 0.31	19.57^d^ ± 0.37
Total tooth volume (cm^3^)	0.249^a^ ± 0.006	0.203^b^ ± 0.004	0.147^c^ ± 0.003	0.113^d^ ± 0.002	0.491^e^ ± 0.023
Total enamel volume (mm^3^)	17.82^a^ ± 1.02	17.88^a^ ± 0.79	12.69^b^ ± 0.54	10.62^b^ ± 0.37	40.77^c^ ± 3.35
Total dentine volume (mm^3^)	161.02^a^ ± 5.09	124.16^b^ ± 3.72	84.31^c^ ± 2.66	63.15^c^ ± 2.24	329.88^d^ ± 17.73
Mean volumetric tooth mineral density (g/cm^3^)	3.054 ± 0.037	2.982 ± 0.041	2.947 ± 0.041	2.959 ± 0.043	2.973 ± 0.028
Volumetric enamel mineral density (μg/mm^3^)	2.402 ± 0.00004	2.467 ± 0.06546	2.402 ± 0.00003	2.402 ± 0.00004	2.402 ± 0.00005
Volumetric dentine mineral density (μg/mm^3^)	2.154^a^ ± 0.031	2.044^b^ ± 0.019	2.048^b^ ± 0.025	2.096^ab^ ± 0.057	2.068^ab^ ± 0.016
Maximum elastic strength (N)	274^a^ ± 15	212^b^ ± 13	169^c^ ± 17	111^d^ ± 17	----
Ultimate force (N)	350^a^ ± 22	266^b^ ± 18	201^c^ ± 15	158^d^ ± 15	957^e^ ± 83

Values are means ± SEM.

^a-e^Mean values that do not share common superscript letter in the same row differ significantly for P < 0.05.

## Discussion

Standard veterinary examination of teeth in sheep would be an essential part of the clinical investigation procedures which enables age estimation or poor productive potential indication [9]. Even though simple clinical dental examination requires relatively small costs, it is less informative and provides basic information on number, size, shape and structure of teeth and their occlusion with dental pad.

This study presents basic morphological, densitometric and mechanical properties of completely developed deciduous teeth such as mandibular incisors, canine and the second premolar in sheep at the age of 5 months of life for the first time. The analyses performed have shown significant differences of weight of teeth between all incisors reaching the highest value for the first incisors and diminishing gradually in i_2_ and i_3_ to the lowest value in i_4_ (canine). The differentiated weight of the teeth resulted from analogical differences of the length and total tooth volume of the incisors. Considering significant difference of weight, length and tooth volume, it is surprising that total enamel volume was comparable in i_1_ and i_2_ as well as in i_3_ and i_4_, even though i_1_ and i_2_ possess approximately 40 – 68% more enamel than i_3_ and i_4_. Gradual reduction of total dentine volume was observed between i_1_ – i_2_, i_2_ – i_3_ and i_3_ – i_4_, that reached 23%, 32% and 25%, respectively. However, densitometric analysis has shown similar values for all the evaluated deciduous teeth in this study when analyzing both MvTMD and vEMD. The only one difference obtained from densitometric evaluation of teeth in sheep has shown significantly higher vDMD in i_1_ when compared to i_3_ and i_4_. Thus, the performed densitometric measurements in both compartments have not shown considerable differences of volumetric tooth mineral density between several deciduous teeth, confirming similar course of mineralization processes of hard dental tissues in all the investigated teeth. Thus, it may be postulated that proper mineral metabolism and mineralization processes during first months of life in sheep lead to comparable mineral density threshold attainment in hard dental tissues of all teeth. The results of densitometric analysis in our study are in accordance to the other reports confirming significantly higher mineral density in enamel than in dentine. It was shown that enamel consists of 95% inorganic substance, 4% water and 1% organic substance, while in dentine all these constituents represent 70%, 20% and 10%, respectively [19-21]. Analogically to results of tooth weight, length and volume, consequent reduction of Wy was observed between i_1_ – i_2_, i_2_ – i_3_ and i_3_ – i_4_ and it reached 23%, 20% and 34%, respectively. Similar decrease of Wf was stated evaluating deciduous incisors and the differences between i_1_ – i_2_, i_2_ – i_3_ and i_3_ – i_4_ reached 24%, 25% and 21%, respectively. In case of the second premolar, its large crown and multi-root anatomical structure contributed to significantly higher values of tooth weight, volume (both in enamel and dentine) and ultimate force when compared to these parameters in incisors and canine. However, tooth length, MvTMD, vEMD and vDMD of p_2_ were similar when compared with the other investigated teeth. It should also be explained here that mechanical analysis of i_1_ – i_4_ and p_2_ was performed with two different methods such as three point bending and compressive tests and thus the assessment of Wy for p_2_ was not possible. Even though the current study was not performed on large animal population, the obtained values of morphological, densitometric and mechanical parameters may be deemed as reference data for deciduous teeth in sheep since the investigated teeth were completely developed and their growth was finished. In case of the other deciduous mandibular teeth that were not evaluated in this study, longer breeding procedure is required to finish their growth and development and receive meaningful data [22].

Precise evaluation of teeth properties in terms of morphology, microstructure and mechanical properties would be much more effective than simple clinical examination to provide data on whole body and calcified tissues metabolism during crucial periods of life. Considering very rapid growth rate during neonatal and postnatal development in sheep, morphometric (both on macro- and microstructural levels), densitometric and mechanical evaluation of deciduous teeth would be informative in terms of physiological, nutritional, pharmacological, toxicological and environmental conditions taking place during first months of life. Experimental studies on growing sheep have shown that advantageous nutritional manipulations during early stages of neonatal development positively influence mineral metabolism and bone tissue properties. Oral administration with alpha-ketoglutarate (AKG) during the first two weeks of life induced improved morphological traits (weight and length), volumetric bone mineral density (vBMD) and mechanical properties (maximum elastic strength and ultimate strength) of ribs in 5 month old ram lambs [23]. Similar effects of neonatal administration with AKG were observed in studies on femur properties, where vBMD of the trabecular and cortical bone compartments and ultimate strength were significantly increased when compared to the controls receiving standard diet [24]. Three week long neonatal administration with *β*-hydroxy-*β*-methylbutyrate (HMB) in lambs significantly increased serum concentrations of bone-specific alkaline phosphatase (BAP) and osteocalcin (OC) – the bone tissue formation markers reflecting osteoblastic activity. Moreover, growth hormone (GH) and insulin-like growth factor-1 (IGF-1), the hormonal factors inducing improved bone tissue metabolism and mineralization, were significantly increased as the consequence of 21-day neonatal administration with HMB. All these effects were associated with permanent changes of bone morphology, mineral density and mechanical endurance. *β*-hydroxy-*β*-methylbutyrate administered in young lambs improved bone weight, length, volume, vBMD of the cortical bone as well as geometrical and mechanical properties of femur and lumbar spine [25]. Even though teeth properties were not evaluated in all these experiments, structural and functional changes of hard dental tissues on macro- and microstructural levels would be expected, especially when one considers significant role of osteoblasts in hard dental tissues formation. Furthermore, previous studies have shown interferences between tooth development and alkaline phosphatase, osteocalcin, bone morphogenetic proteins (BMP) and bone sialoproteins involved in signaling functions and mediating hard dental tissue interactions during development [2,26].

## Conclusions

An advantage resulting from morphometric, densitometric and mechanical dental evaluation in animals is the fact that the methodological approach is noninvasive since the deciduous teeth undergo physiological replacement with permanent teeth, taking place at several stages and does not require substantial intervention, sedation or general anesthesia, when compared to *in vivo* determination of skeletal system properties. Furthermore, *in vivo* evaluation of bones in sheep with microtomography and mechanical tests is not possible due to limitations resulting from ability to scan small samples in microtomographic devices, as well as consideration of ethical issues [27,28]. These limitations do not concern deciduous teeth which can be easy collected for analyses from large animal population and reflect mineral status and metabolism resulting from postnatal growth and development of the whole flock. In individual cases, evaluation of properties of deciduous teeth may serve for breeding selection and further reproduction of sheep possessing favorable traits of teeth and better masticatory system functions, leading to improved performance and economic efficiency of the flock. Thus, results from morphometric, densitometric and mechanical evaluation of the teeth may indicate optimal breeding direction of growing sheep.

## Abbreviations

MIH: Molar incisor hypomineralization; EDJ: Enamel-dentin-junction; i1: First incisor; i2: Second incisor; i3: Third incisor; i4: Fourth incisor (canine); p2: Second premolar; QCT: Quantitative computed tomography; MvTMD: Volumetric tooth mineral density; Tvol: Total tooth volume; HU: Hounsfield units; μCT: Microtomography; Evol: Total enamel volume; vEMD: Volumetric enamel mineral density; vDMD: Volumetric dentine mineral density; Dvol: Total dentine volume; ROI: Region of interest; Wy: Maximum elastic strength; Wf: Ultimate force; AKG: Alpha-ketoglutarate; HMB: *β*-hydroxy-*β*-methylbutyrate; BAP: Bone-specific alkaline phosphatase; OC: Osteocalcin; GH: Growth hormone; IGF-1: Insulin-like growth factor-1; BMP: Bone morphogenetic proteins

## Competing interest

The authors declare that they have no competing interests.

## Authors’ contributions

MRT was responsible for the concept of the study, supervised all stages of the study, participated in animal management, collection and interpretation of data, as well as writing the manuscript. AS was responsible for the concept of the study, collection and interpretation of data from mechanical analyses, statistical analysis and writing the manuscript. WK performed QCT analysis of teeth and interpretation of data and supervised all stages of the study. BT was responsible for references collection, interpretation of data and writing the manuscript. IŁ-S was responsible for teeth preparation, interpretation of data and writing the manuscript. JB was responsible for micro CT analysis of teeth, participated in interpretation of data and critical revision of the manuscript. MO was responsible for micro CT analysis of teeth and participated in interpretation of data. All authors have read and approved the manuscript.

## References

[B1] Kierdorf H Witzel C Upex B Dobney K Kierdorf U Enamel hypoplasia in molars of sheep and goats, and its relationship to the pattern of tooth crown growth J Anat 2012 220 484 495 10.1111/j.1469-7580.2012.01482.x 22352403 PMC3403278

[B2] Kmieć Z Histology and cytophysiology tooth and mouth 2007 Elsevier Urban & Partner: Wrocław

[B3] Erjavec V Crossley D Initial observations of cheek tooth abnormalities in sheep in Slovenia Vet Rec 2010 167 134 137 10.1136/vr.c3328 20656992

[B4] Wiese SC Pethick DW Milton JTB Davidson RH McIntyre BL D’Souza DN Effect of teeth eruption on growth performance and meat quality of sheep A J E A 2005 45 509 515 10.1071/EA03258

[B5] Baker GJ Easley J Baker GJ, Easley J The systemic effects of dental disease Equine Dentistry 1999 London: W. B. Saunders 127 138

[B6] Crossley DA Miguélez MM Skull size and cheek-tooth length in wild-caught and captive-bred chinchillas Arch Oral Biol 2001 46 919 928 10.1016/S0003-9969(01)00055-3 11451406

[B7] Bloxham GP Purton DG Demineralisation and incisor wear: an in vitro study N Z J Agric Res 1991 34 277 279 10.1080/00288233.1991.10417665

[B8] Healy WB Cutress TW Michie C Wear of sheep's teeth. IV. Reduction of soil ingestion and tooth wear by supplementary feeding N Z J Agric Res 1967 10 201 209 10.1080/00288233.1967.10425127

[B9] Ridler A West D Examination of teeth in sheep health management Small Rumin Res 2010 92 92 95 10.1016/j.smallrumres.2010.04.014

[B10] Spence J Aitchison G Clinical aspects of dental disease in sheep In Pract 1986 8 128 135 10.1136/inpract.8.4.128

[B11] Fagrell TG Salmon P Melin L Norén JG Onset of molar incisor hypomineralization (MIH) Swed Dent J 2013 37 61 70 23957140

[B12] West DM Dental disease of sheep N Z Vet J 2002 50 102 104 10.1080/00480169.2002.36282 21838636

[B13] Boddie GF Fluorosis in domestic animals Vet Rec 1947 59 301 303 20251207

[B14] Franklin MC Diet and dental development in sheep 1950 Melbourne: Commonwealth Scientific and Industrial Research Organisation. Bulletin 252

[B15] Milhaud G Borba M Krishnaswamy S Effect of fluoride ingestion on dental fluorosis in sheep Am J Vet Res 1987 48 873 879 3592393

[B16] Suckling GW Defects of enamel of sheep resulting from trauma during tooth development J Dent Res 1980 59 1541 1548 10.1177/00220345800590092701 6931141

[B17] Suckling GW Elliott DC Thurley DC The production of developmental defects of enamel in the incisor teeth of penned sheep resulting from induced parasitism Arch Oral Biol 1983 28 393 399 10.1016/0003-9969(83)90134-6 6578757

[B18] Tymczyna B Tatara MR Krupski W Tymczyna-Sobotka M Bachanek T Interrelationships between tooth properties and biochemical bone turnover markers investigated on six-month old pig model J Vet Med Sci 2013 75 269 274 10.1292/jvms.12-0394 23076035

[B19] Marshall GW Jr Marshall SJ Kinney JH Balooch M The dentin substrate: structure and properties related to bonding J Dent 1997 25 441 458 10.1016/S0300-5712(96)00065-6 9604576

[B20] Wong FS Anderson P Fan H Davis GR X-ray microtomographic study of mineral concentration distribution in deciduous enamel Arch Oral Biol 2004 49 937 944 10.1016/j.archoralbio.2004.05.011 15353251

[B21] Zou W Hunter N Swain MV Application of polychromatic μCT for mineral density determination J Dent Res 2011 90 18 30 10.1177/0022034510378429 20858779 PMC3144098

[B22] Cocquyt G Driessen B Simoens P Variability in the eruption of the permanent incisor teeth in sheep Vet Rec 2005 157 619 623 16284330 10.1136/vr.157.20.619

[B23] Tatara MR Tygesen MP Sawa-Wojtanowicz B Krupski W Majcher P Harrison AP Bone development: the effect of short term alpha-ketoglutarate administration on long term mechanical properties of ribs in ram lambs Small Rumin Res 2007 67 179 183 10.1016/j.smallrumres.2005.09.031

[B24] Harrison AP Tygesen MP Sawa-Wojtanowicz B Husted S Tatara MR α-Ketoglutarate treatment early in postnatal life improves bone density in lambs at slaughter Bone 2004 35 204 209 10.1016/j.bone.2004.03.016 15207758

[B25] Tatara MR Neonatal programming of skeletal development in sheep is mediated by somatotrophic axis function Exp Physiol 2008 93 763 772 10.1113/expphysiol.2007.041145 18263656

[B26] Cobourne MT The genetic control of early odontogenesis Br J Orthod 1999 26 21 28 10.1093/ortho/26.1.21 10333884

[B27] Badea CT Drangova M Holdsworth DW Johnson GA In vivo small animal imaging using micro-CT and digital subtraction angiography Phys Med Biol 2008 53 R319 R350 10.1088/0031-9155/53/19/R01 18758005 PMC2663796

[B28] Schladitz K Quantitative micro-CT J Microsc 2011 243 111 117 10.1111/j.1365-2818.2011.03513.x 21762162

